# Crystal structure of a mixed-ligand terbium(III) coordination polymer containing oxalate and formate ligands, having a three-dimensional fcu topology

**DOI:** 10.1107/S205698901502397X

**Published:** 2016-01-01

**Authors:** Chainok Kittipong, Phailyn Khemthong, Filip Kielar, Yan Zhou

**Affiliations:** a Department of Physics, Faculty of Science and Technology, Thammasat University, Khlong Luang, Pathum Thani, 12120, Thailand; b Department of Chemistry, Faculty of Science, Naresuan University, Muang, Phitsanulok, 65000, Thailand; c Department of Chemistry, The Hong Kong University of Science and Technology, Clear Water Bay, Kowloon, Hong Kong

**Keywords:** coordination polymers, crystal structure, lanthanide, luminescence, terbium(III)

## Abstract

The title compound, poly[(*μ*_3_-formato)(*μ*_4_-oxalato)terbium(III)], [Tb(CHO_2_)(C_2_O_4_)]_*n*_, is a three-dimensional coordination polymer, and is isotypic with the La^III^, Ce^III^ and Sm^III^ analogues. The asymmetric unit contains one Tb^III^ ion, one formate anion (CHO_2_^−^) and half of an oxalate anion (C_2_O_4_^2−^), the latter being completed by application of inversion symmetry. The Tb^III^ ion is nine-coordinated in a distorted tricapped trigonal–prismatic manner by two chelating carboxyl­ate groups from two C_2_O_4_^2−^ ligands, two carboxyl­ate oxygen atoms from another two C_2_O_4_^2−^ ligands and three oxygen atoms from three CHO_2_^−^ ligands, with the Tb—O bond lengths and the O—Tb—O bond angles ranging from 2.4165 (19) to 2.478 (3) Å and 64.53 (6) to 144.49 (4)°, respectively. The CHO_2_^−^ and C_2_O_4_^2−^ anions adopt *μ*_3_-bridging and *μ*_4_-chelating-bridging coordination modes, respectively, linking adjacent Tb^III^ ions into a three-dimensional 12-connected fcu topology with point symbol (3^24^.4^36^.5^6^). The title compound exhibits thermal stability up to 623 K, and also displays strong green photoluminescence in the solid state at room temperature.

## Abstract 2

The crystal structure of *catena*-[(*μ*_3_-formato)(*μ*_4_-oxalato)terbium(III)] features a three-dimensional 12-connected fcu topology with point symbol (3^24^.4^36^.5^6^), exhibiting thermal stability up to 623 K and strong green photoluminescence in the solid state at room temperature.

## Chemical context   

Owing to their high colour purity, high luminescence quantum yields, narrow bandwidths, relatively long lifetimes and large Stokes shifts arising from 4*f* orbitals, coordination polymers of lanthanide(III) ions and organic linker ligands have received much attention from chemists during the past decade for the development of fluorescent probes and electroluminescent devices (Hasegawa & Nakanishi, 2015 ▸). In particular, polymeric Eu^III^ and Tb^III^ compounds with a range of organic linker ligands are the most intense emitters among the lanthanide(III) series, and they have been developed extensively as ion sensing and optical materials (Cui *et al.*, 2014 ▸). Lanthan­ide(III) ions are known to have a high affinity and preference for hard donor atoms. Thus, di­carb­oxy­lic acid ligands containing aliphatic, aromatic and *N*-heterocyclic moieties have been widely employed in the construction of luminescent lanthanide coordination polymers (So *et al.*, 2015 ▸). Among the ligands in this class, for instance, terephthalic acid is known to provide an efficient energy transfer to support strong lanthan­ide(III)-centered luminescent emission *via* the *‘*antenna effect*’* (Samuel *et al.*, 2009 ▸). On the other hand, small rigid planar species with versatile coordination oxygen donor sites such as oxalate, carbonate, nitrate, and formate anions are also a very important class of ligands for the preparation of lanthanide coordination polymers (Hong *et al.*, 2014 ▸; Gupta *et al.*, 2015 ▸). These small versatile ligands can bind to metals in different modes, resulting in the formation of multi-dimensional coordination networks with short inter­metallic distances, which can aid the energy-transfer process between chromophoric antenna ligands and lanthanide(III) ions (Wang *et al.*, 2012 ▸). In addition, the oxalate anion has proved to be an efficient sensitizer for lanthanide(III)-based emission (Cheng *et al.*, 2007 ▸). Recently, many multi-dimensional luminescent lanthanide coordination polymers containing antenna and small rigid planar mixed ligands have been reported (Xu *et al.*, 2013 ▸; Wang *et al.*, 2013 ▸). However, only a few compounds with mixed small rigid planar ligands alone have been described in the literature (Zhang *et al.*, 2007 ▸; Huang *et al.*, 2013 ▸; Tang *et al.*, 2014 ▸).
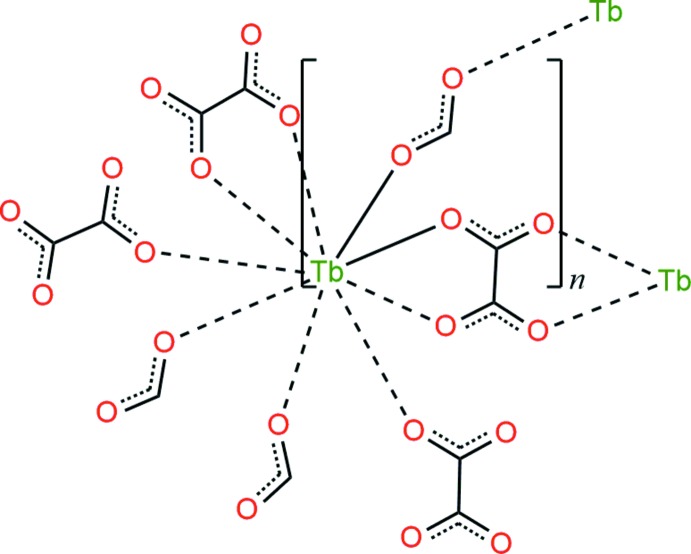


Herein, we report the synthesis and structure of a terbium(III) coordination polymer containing formate and oxalate mixed ligands, [Tb(CHO_2_)(C_2_O_4_)]_*n*_, (I), having a three-dimensional 12-connected fcu topology with point symbol (3^24^.4^36^.5^6^). The thermal stability and luminescent properties of compound (I) have also been investigated.

## Structural commentary   

**Figure 1:**
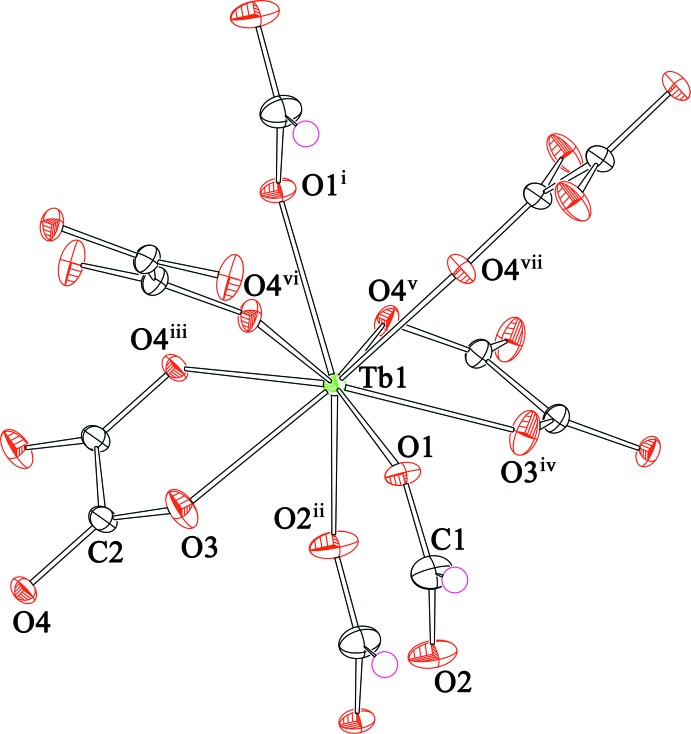
Coordination environment of the Tb^III^ ion in (I). Displacement ellipsoids are drawn at the 50% probability level and H atoms are shown as small spheres of arbitrary radii. For symmetry codes, see Table 1 ▸.

**Figure 2:**
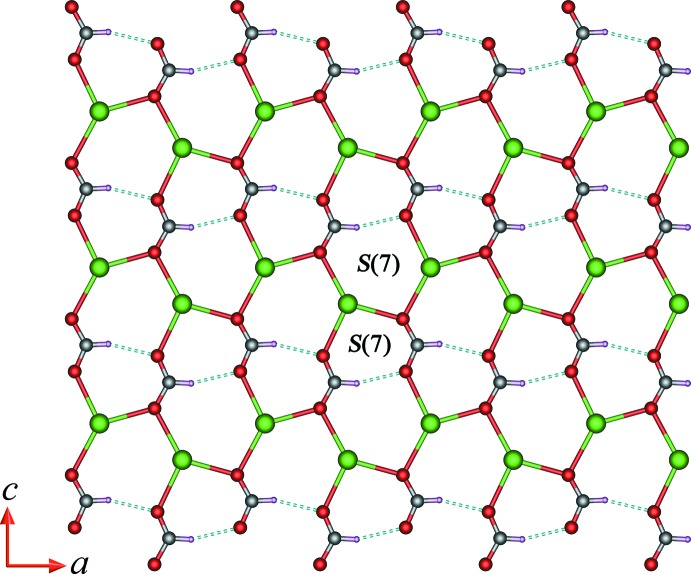
A view of the two-dimensional terbium-formate network in (I), showing the monolayer structure projected in the *ac* plane. The dashed lines indicate the intra­layer C—H⋯O hydrogen bonds (Table 2 ▸).

Single crystal X-ray diffraction analysis revealed that (I) is isotypic in the ortho­rhom­bic *Pnma* space group with the La^III^, Ce^III^ and Sm^III^ analogues (Romero *et al.*, 1996 ▸). The asymmetric unit contains one Tb^III^ ion, one formate anion, and half of an oxalate anion. As shown in Fig. 1 ▸, each Tb^III^ ion is nine-coordinated in a distorted tricapped trigonal prismatic manner (Fig. 1 ▸) by two chelating carboxyl­ate groups from two oxalate ligands, two carboxyl­ate oxygen atoms from another two oxalate ligands and three oxygen atoms from three formate ligands, with the O—Tb—O bond angles ranging from 64.53 (6) to 144.49 (4)°. The Tb—O bond lengths in (I) are in the range of 2.4165 (19) to 2.478 (3) Å (Table 1 ▸), which is in good agreement with the reported distances for other Tb^III^ complexes containing oxygen donor ligands (Cheng *et al.*, 2007 ▸; Zhu *et al.*, 2007 ▸). All of the bond lengths and bond angles in the formate and oxalate anions are also within normal ranges (Rossin *et al.*, 2012 ▸; Hong *et al.*, 2014 ▸; Gupta *et al.*, 2015 ▸). The coordination modes of the formate and oxalate ligands in (I) (Fig. 2 ▸) are commonly observed in lanthanide coordination polymers (Zhang *et al.*, 2007 ▸; Rossin *et al.*, 2012 ▸).

**Figure 3:**
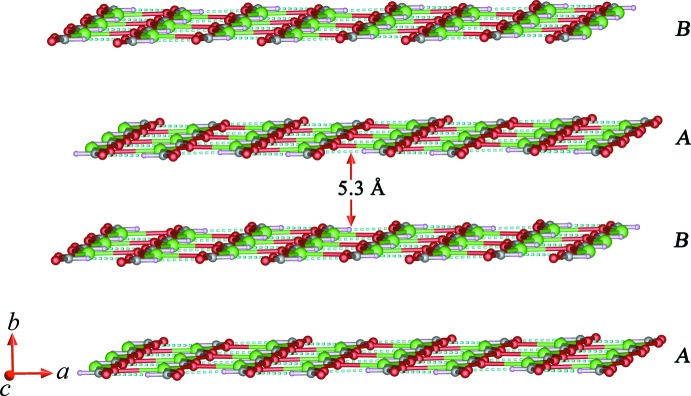
The terbium-formate layered structure viewed along the *c* axis.

**Figure 4:**
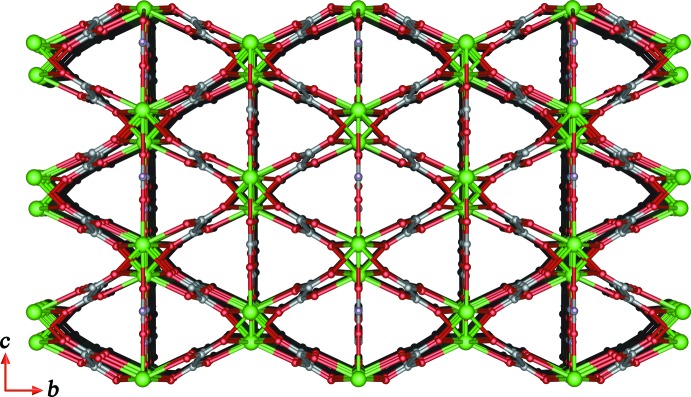
A perspective view along the *a* axis of the three-dimensional framework.

**Figure 5:**
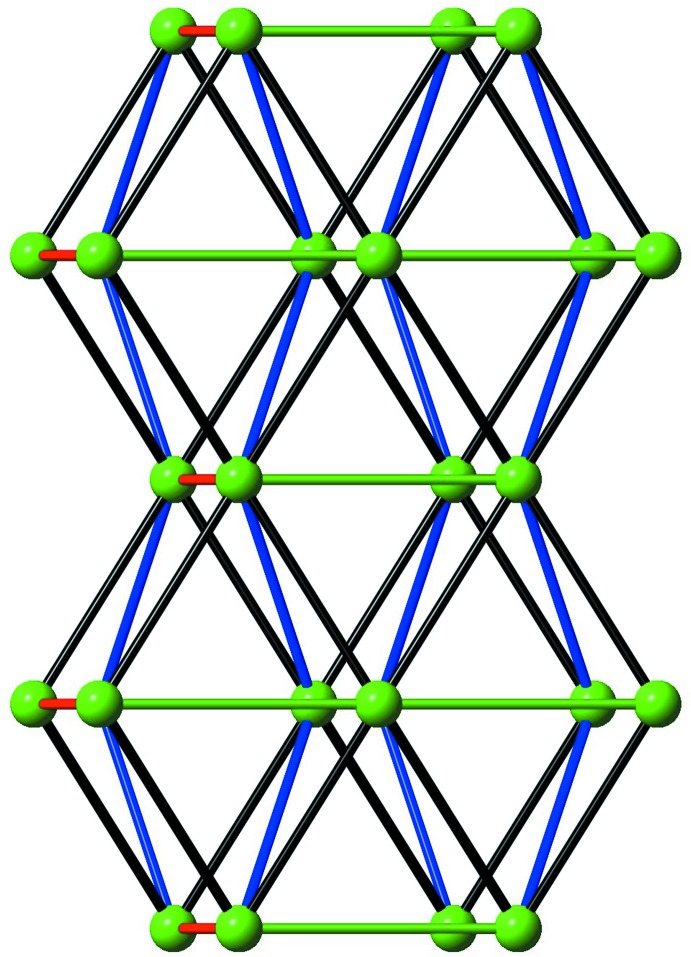
Schematic representation of the 12-connected fcu topology in (I).

As shown in Fig. 2 ▸, each formate anion adopts a μ_3_-bridging coordination mode connecting three Tb^III^ ions, forming a two-dimensional (2-D) layer in the *ac* plane. In the 2-D terbium-formate monolayer, the Tb1⋯Tb1 separations along the formate ligands in *syn*–*anti* and *anti*–*anti* O1,O2-bridging coordination modes (Rossin *et al.*, 2012 ▸) are 6.1567 (3) and 6.6021 (2) Å, respectively. The adjacent 2-D monolayers are stacked in an –*ABA*– sequence running perpendicular to the *b* axis with an inter­layer spacing of *ca* 5.3 Å (Fig. 3 ▸). The oxalate ligand adopts a *μ*_4_-chelating-bridging coordination mode, linking four Tb^III^ ions along the *a* axis to form a three-dimensional (3-D) terbium–oxalate open framework (Fig. 3 ▸). The Tb1⋯Tb1 distance *via* the formate O1- and oxalate O4-bridging ligands is 3.8309 (2) Å with the Tb1—O1—Tb1 and Tb1—O4—Tb1 bond angles being 103.00 (9) and 102.79 (6)°, respectively. On the other hand, the channels in the 3-D open framework have an approximate rhombic shape with a Tb1⋯Tb1 separation of 6.2670 (2) Å, and are cross-linked parallel to the *c* axis by bridging formate ligands as shown in Fig. 4 ▸. The presence of guest mol­ecules in the lattice as well as the formation of inter­penetrated networks of (I) are thus prevented. Furthermore, the topology of the network in (I) was analysed using *TOPOS* (Blatov *et al.*, 2000 ▸). As schematically depicted in Fig. 5 ▸, the overall framework can be defined as a 12-connected fcu topology with point symbol (3^24^.4^36^.5^6^) by linking each adjacent layer of Tb^III^ atoms *via* formate and oxalate ligands.

The infrared spectrum of (I) was collected from a polycrystalline sample pelletized with KBr, in the range 4000–400 cm^−1^. This spectrum indicates the presence of the carboxyl­ate groups of the ligands by appearance of the strong absorption bands at 1630 and 1315 cm^−1^ for the asymmetric (ν_asym_COO^−^) and the symmetric (ν_sym_COO^−^) carboxyl­ate vibrations, respectively (Deacon & Phillips, 1980 ▸). To examine the thermal stability of (I), thermogravimetric analysis was performed on a polycrystalline sample under a nitro­gen atmosphere in the temperature range of 303–1273 K. There is no weight loss before 623 K due to the stability of the fcu-type 3-D frameworks. The decomposition of the framework, however, occurred rapidly at temperatures above 628 K.

**Figure 6:**
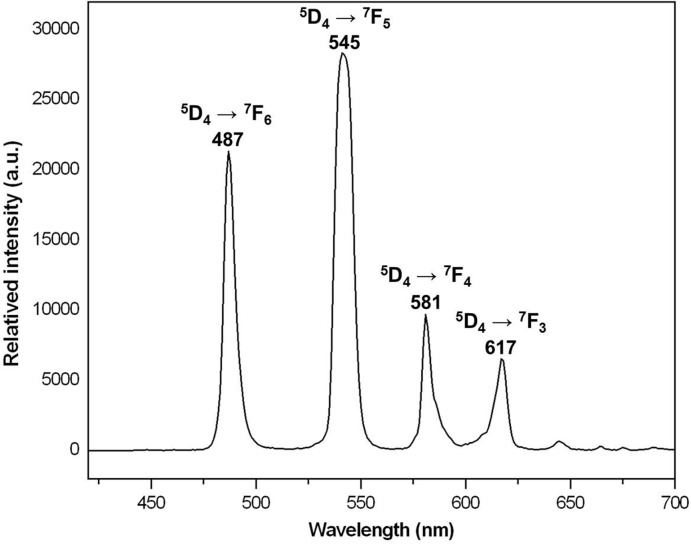
The solid-state emission spectrum of (I) at room temperature.

The photoluminescence properties of (I) were investigated in the solid state at room temperature. The emission spectrum is shown in Fig. 6 ▸. The emission spectrum upon excitation at 305 nm exhibits the characteristic *f–f* transitions of Tb^III^ ions (Bünzli, 2010 ▸). The emission peaks at 487, 543, 585, and 617 nm can be assigned to the ^5^ *D*_4_ → ^7^ *F_J_* (*J* = 6, 5, 4, 3) transitions, respectively. The most intense transition is ^5^ *D*_4_ → ^7^ *F*_5_, which implies the emitted light is green. The emission lifetime of (I) is 1.79 ms.

## Supra­molecular features   

The two-dimensional terbium-formate monolayers are stabilized by weak intra-layer C1—H1⋯O2^viii^ hydrogen bonds giving *S*(7) graph-set motifs (Bernstein *et al.*, 1995 ▸), in which each formate anion acts as a donor and acceptor for one hydrogen bond (Table 2 ▸, Fig. 2 ▸).

## Database survey   

A search of the Cambridge Structural Database (Groom & Allen, 2014 ▸) for lanthanide coordination polymers containing mixed oxalate and formate ligands gave four hits (RIFQIG, RIFRED, RIFRIH; Romero *et al.*, 1996 ▸; RIFQIG01; Tan *et al.*, 2009 ▸), which are isotypic with the title compound (I) as previously mentioned. The structures involving oxalate and acetate analogues have also been reported (AZOCIC; Di *et al.*, 2011 ▸; Gutkowski *et al.*, 2011 ▸; SOPPIX; Zhang *et al.*, 2009 ▸; VORBUA; Koner & Goldberg, 2009 ▸).

## Synthesis and crystallization   

All reagents were of analytical grade and were used as obtained from commercial sources without further purification. Synthesis of (I): TbCl_3_·6H_2_O (0.187 g, 0.5 mmol), oxalic acid (0.045 g, 0.5 mmol), Na_2_CO_3_ (0.011 g, 0.1 mmol), and a mixture (1:1 *v*/*v*) of *N*,*N*′-di­methyl­formamide (DMF) and water (6 ml) was sealed in a 23 ml Teflon-lined stainless steel vessel and heated under autogenous pressure at 463 K for two days. After the reactor was cooled to room temperature, colorless block-shaped crystals were filtered off and dried in air. Yield: 0.118 g (63% based on the Tb^III^ source). Analysis (%) calculated for C_3_HO_6_Tb (291.96): C, 12.34; H, 0.35%. Found: C, 12.40; H, 0.33%. IR (KBr, cm^−1^): 2823 (*w*), 2491 (*w*), 1630 (*s*), 1440 (*w*), 1315 (*s*), 1022 (*m*), 914 (*w*), 795 (*s*), 611 (*w*), 492 (*s*), 408 (*w*).

## Refinement   

Crystal data, data collection and structure refinement details are summarized in Table 3 ▸. The formate H atom was found in a difference electron-density map and was refined using a riding-model approximation, with C—H = 0.93 Å and with *U*_iso_(H) = 1.2*U*_eq_(C).

## Supplementary Material

Crystal structure: contains datablock(s) I. DOI: 10.1107/S205698901502397X/zs2356sup1.cif

Structure factors: contains datablock(s) I. DOI: 10.1107/S205698901502397X/zs2356Isup2.hkl

Supporting information file. DOI: 10.1107/S205698901502397X/zs2356Isup3.cdx

Supporting information file. DOI: 10.1107/S205698901502397X/zs2356Isup4.docx

CCDC reference: 1436132

Additional supporting information:  crystallographic information; 3D view; checkCIF report

## Figures and Tables

**Table 1:** Selected bond lengths (Å)

Tb1—O1	2.417 (3)	Tb1—O4^iv^	2.4370 (18)
Tb1—O1^i^	2.478 (3)	Tb1—O4^v^	2.4651 (17)
Tb1—O2^ii^	2.437 (3)	Tb1—O4^vi^	2.4370 (17)
Tb1—O3^iii^	2.4165 (19)	Tb1—O4^vii^	2.4651 (17)
Tb1—O3	2.4165 (19)		

Symmetry codes: (i) 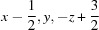; (ii) 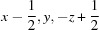; (iii) 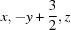; (iv) 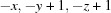; (v) 
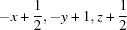
; (vi) 
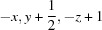
; (vii) 
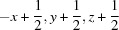
.

**Table 2:** Hydrogen-bond geometry (Å, °)

*D*—H⋯*A*	*D*—H	H⋯*A*	*D*⋯*A*	*D*—H⋯*A*
C1—H1⋯O2^viii^	0.93	2.15	3.051 (5)	164

Symmetry code: (viii) 
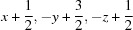
.

**Table 3:** Experimental details

Crystal data	
Chemical formula	[Tb(CHO_2_)(C_2_O_4_)]
*M*_r_	291.96
Crystal system, space group	Orthorhombic, *P* *n* *m* *a*
Temperature (K)	296
*a*, *b*, *c* (Å)	7.0138 (3), 10.6077 (4), 6.6021 (2)
*V* (Å^3^)	491.20 (3)
*Z*	4
Radiation type	Mo *K*α
μ (mm^−1^)	14.36
Crystal size (mm)	0.2 × 0.12 × 0.08

Data collection	
Diffractometer	Bruker D8 QUEST CMOS
Absorption correction	Multi-scan (*SADABS*; Bruker, 2014 ▸)
*T*_min_, *T*_max_	0.655, 0.746
No. of measured, independent and observed [*I* > 2σ(*I*)] reflections	6517, 638, 594
*R*_int_	0.028
(sin θ/λ)_max_ (Å^−1^)	0.666

Refinement	
*R*[*F* ^2^ > 2σ(*F* ^2^)], *wR*(*F* ^2^), *S*	0.012, 0.025, 1.10
No. of reflections	638
No. of parameters	52
H-atom treatment	H-atom parameters constrained
Δρ_max_, Δρ_min_ (e Å^−3^)	0.75, −0.63

Computer programs: *APEX2* and *SAINT* (Bruker, 2014 ▸), *SHELXS2014* (Sheldrick, 2008 ▸), *SHELXL2014* (Sheldrick, 2015 ▸), *OLEX2* (Dolomanov *et al.*, 2009 ▸), *publCIF* (Westrip, 2010 ▸) and *enCIFer* (Allen *et al.*, 2004 ▸).

## supplementary crystallographic information

### Crystal data

**Table:**

[Tb(CHO_2_)(C_2_O_4_)]	*D*_x_ = 3.948 Mg m^−^^3^
*M**_r_* = 291.96	Mo *K*α radiation, λ = 0.71073 Å
Orthorhombic, *P**n**m**a*	Cell parameters from 3952 reflections
*a* = 7.0138 (3) Å	θ = 3.6–28.3°
*b* = 10.6077 (4) Å	µ = 14.36 mm^−^^1^
*c* = 6.6021 (2) Å	*T* = 296 K
*V* = 491.20 (3) Å^3^	Block, colourless
*Z* = 4	0.2 × 0.12 × 0.08 mm
*F*(000) = 528	

### Data collection

**Table:**

Bruker D8 QUEST CMOS diffractometer	638 independent reflections
Radiation source: microfocus sealed x-ray tube, Incoatec Iµus	594 reflections with *I* > 2σ(*I*)
Graphite Double Bounce Multilayer Mirror monochromator	*R*_int_ = 0.028
Detector resolution: 10.5 pixels mm^-1^	θ_max_ = 28.3°, θ_min_ = 3.6°
ω and φ scans	*h* = −9→9
Absorption correction: multi-scan (*SADABS*; Bruker, 2014)	*k* = −13→14
*T*_min_ = 0.655, *T*_max_ = 0.746	*l* = −8→8
6517 measured reflections	

### Refinement

**Table:**

Refinement on *F*^2^	Primary atom site location: structure-invariant direct methods
Least-squares matrix: full	Secondary atom site location: difference Fourier map
*R*[*F*^2^ > 2σ(*F*^2^)] = 0.012	Hydrogen site location: inferred from neighbouring sites
*wR*(*F*^2^) = 0.025	H-atom parameters constrained
*S* = 1.10	*w* = 1/[σ^2^(*F*_o_^2^) + (0.0092*P*)^2^ + 0.8666*P*] where *P* = (*F*_o_^2^ + 2*F*_c_^2^)/3
638 reflections	(Δ/σ)_max_ = 0.001
52 parameters	Δρ_max_ = 0.75 e Å^−^^3^
0 restraints	Δρ_min_ = −0.63 e Å^−^^3^

### Special details

**Table:**

Experimental. *SADABS2014* (Bruker, 2014) was used for absorption correction. wR2(int) was 0.0566 before and 0.0416 after correction. The ratio of minimum to maximum transmission is 0.8789. The λ/2 correction factor is 0.00150.
Geometry. All e.s.d.'s (except the e.s.d. in the dihedral angle between two l.s. planes) are estimated using the full covariance matrix. The cell e.s.d.'s are taken into account individually in the estimation of e.s.d.'s in distances, angles and torsion angles; correlations between e.s.d.'s in cell parameters are only used when they are defined by crystal symmetry. An approximate (isotropic) treatment of cell e.s.d.'s is used for estimating e.s.d.'s involving l.s. planes.
Refinement. Refinement of *F*^2^ against ALL reflections. The weighted *R*-factor *wR* and goodness of fit *S* are based on *F*^2^, conventional *R*-factors *R* are based on *F*, with *F* set to zero for negative *F*^2^. The threshold expression of *F*^2^ > σ(*F*^2^) is used only for calculating *R*-factors(gt) *etc*. and is not relevant to the choice of reflections for refinement. *R*-factors based on *F*^2^ are statistically about twice as large as those based on *F*, and *R*- factors based on ALL data will be even larger.

### Fractional atomic coordinates and isotropic or equivalent isotropic displacement parameters (Å^2^)

**Table:**

	*x*	*y*	*z*	*U*_iso_*/*U*_eq_	
Tb1	0.20226 (2)	0.7500	0.63323 (2)	0.00749 (6)	
O1	0.5347 (4)	0.7500	0.5364 (4)	0.0132 (5)	
O2	0.5527 (4)	0.7500	0.2000 (4)	0.0237 (7)	
O3	0.2384 (3)	0.54490 (18)	0.4786 (3)	0.0186 (4)	
O4	0.0873 (3)	0.37671 (16)	0.3522 (3)	0.0120 (4)	
C1	0.6227 (6)	0.7500	0.3693 (6)	0.0197 (8)	
H1	0.7551	0.7500	0.3761	0.024*	
C2	0.0956 (4)	0.4788 (2)	0.4518 (4)	0.0124 (5)	

### Atomic displacement parameters (Å^2^)

**Table:**

	*U*^11^	*U*^22^	*U*^33^	*U*^12^	*U*^13^	*U*^23^
Tb1	0.00799 (8)	0.00750 (8)	0.00698 (9)	0.000	−0.00049 (7)	0.000
O1	0.0110 (13)	0.0209 (14)	0.0076 (13)	0.000	0.0013 (10)	0.000
O2	0.0235 (16)	0.0381 (18)	0.0096 (14)	0.000	−0.0010 (12)	0.000
O3	0.0133 (9)	0.0143 (9)	0.0282 (11)	−0.0028 (7)	0.0010 (8)	−0.0093 (9)
O4	0.0136 (8)	0.0093 (8)	0.0131 (9)	−0.0011 (7)	0.0027 (7)	−0.0041 (7)
C1	0.0141 (17)	0.029 (2)	0.016 (2)	0.000	−0.0009 (16)	0.000
C2	0.0151 (12)	0.0112 (11)	0.0109 (12)	0.0003 (10)	−0.0002 (10)	−0.0020 (10)

### Geometric parameters (Å, º)

**Table:**

Tb1—O1	2.417 (3)	Tb1—O4^vii^	2.4651 (17)
Tb1—O1^i^	2.478 (3)	O1—C1	1.265 (5)
Tb1—O2^ii^	2.437 (3)	O2—C1	1.221 (5)
Tb1—O3^iii^	2.4165 (19)	O3—C2	1.235 (3)
Tb1—O3	2.4165 (19)	O4—C2	1.268 (3)
Tb1—O4^iv^	2.4370 (18)	C1—H1	0.9300
Tb1—O4^v^	2.4651 (17)	C2—C2^iv^	1.551 (5)
Tb1—O4^vi^	2.4370 (17)		
			
Tb1^viii^—Tb1—Tb1^i^	132.533 (9)	O4^vi^—Tb1—Tb1^viii^	138.32 (4)
O1—Tb1—Tb1^viii^	39.06 (6)	O4^v^—Tb1—Tb1^viii^	38.34 (4)
O1^i^—Tb1—Tb1^i^	37.94 (6)	O4^vi^—Tb1—Tb1^i^	38.87 (4)
O1—Tb1—Tb1^i^	171.60 (6)	O4^vii^—Tb1—Tb1^i^	108.19 (4)
O1^i^—Tb1—Tb1^viii^	94.59 (6)	O4^iv^—Tb1—Tb1^i^	38.87 (4)
O1—Tb1—O1^i^	133.65 (7)	O4^iv^—Tb1—Tb1^viii^	138.33 (4)
O1—Tb1—O2^ii^	100.16 (9)	O4^v^—Tb1—Tb1^i^	108.19 (4)
O1—Tb1—O4^vii^	65.01 (6)	O4^vii^—Tb1—Tb1^viii^	38.34 (4)
O1—Tb1—O4^vi^	144.49 (4)	O4^iv^—Tb1—O1^i^	64.53 (6)
O1—Tb1—O4^v^	65.01 (6)	O4^vii^—Tb1—O1^i^	76.57 (6)
O1—Tb1—O4^iv^	144.49 (4)	O4^vi^—Tb1—O1^i^	64.53 (6)
O2^ii^—Tb1—Tb1^viii^	139.22 (7)	O4^v^—Tb1—O1^i^	76.57 (6)
O2^ii^—Tb1—Tb1^i^	88.25 (7)	O4^vi^—Tb1—O2^ii^	71.16 (7)
O2^ii^—Tb1—O1^i^	126.19 (9)	O4^iv^—Tb1—O2^ii^	71.16 (7)
O2^ii^—Tb1—O4^v^	141.92 (5)	O4^v^—Tb1—O4^vii^	66.08 (8)
O2^ii^—Tb1—O4^vii^	141.92 (5)	O4^vi^—Tb1—O4^v^	140.95 (3)
O3—Tb1—Tb1^viii^	94.25 (5)	O4^iv^—Tb1—O4^vi^	66.94 (8)
O3^iii^—Tb1—Tb1^i^	105.42 (5)	O4^iv^—Tb1—O4^v^	100.09 (6)
O3^iii^—Tb1—Tb1^viii^	94.25 (5)	O4^vi^—Tb1—O4^vii^	100.09 (6)
O3—Tb1—Tb1^i^	105.42 (5)	O4^iv^—Tb1—O4^vii^	140.95 (3)
O3—Tb1—O1	77.72 (5)	Tb1—O1—Tb1^viii^	103.00 (9)
O3—Tb1—O1^i^	114.93 (5)	C1—O1—Tb1^viii^	122.4 (2)
O3^iii^—Tb1—O1^i^	114.93 (5)	C1—O1—Tb1	134.6 (2)
O3^iii^—Tb1—O1	77.72 (5)	C1—O2—Tb1^ix^	130.8 (3)
O3—Tb1—O2^ii^	70.35 (5)	C2—O3—Tb1	119.13 (17)
O3^iii^—Tb1—O2^ii^	70.35 (5)	Tb1^iv^—O4—Tb1^x^	102.79 (6)
O3—Tb1—O3^iii^	128.40 (10)	C2—O4—Tb1^x^	137.90 (16)
O3—Tb1—O4^vii^	132.53 (6)	C2—O4—Tb1^iv^	119.27 (16)
O3—Tb1—O4^vi^	126.90 (6)	O1—C1—H1	116.5
O3^iii^—Tb1—O4^iv^	126.90 (6)	O2—C1—O1	127.1 (4)
O3^iii^—Tb1—O4^vi^	66.88 (6)	O2—C1—H1	116.5
O3^iii^—Tb1—O4^v^	132.52 (6)	O3—C2—O4	126.6 (2)
O3^iii^—Tb1—O4^vii^	72.19 (6)	O3—C2—C2^iv^	118.5 (3)
O3—Tb1—O4^v^	72.19 (6)	O4—C2—C2^iv^	114.9 (3)
O3—Tb1—O4^iv^	66.88 (6)		
			
Tb1^viii^—Tb1—O1—C1	180.0	O2^ii^—Tb1—O3—C2	−67.5 (2)
Tb1^i^—Tb1—O3—C2	14.9 (2)	O3^iii^—Tb1—O1—Tb1^viii^	112.87 (5)
Tb1^viii^—Tb1—O3—C2	151.3 (2)	O3—Tb1—O1—Tb1^viii^	−112.87 (5)
Tb1—O1—C1—O2	0.0	O3^iii^—Tb1—O1—C1	−67.13 (5)
Tb1^viii^—O1—C1—O2	180.0	O3—Tb1—O1—C1	67.13 (5)
Tb1^ix^—O2—C1—O1	180.0	O3^iii^—Tb1—O3—C2	−109.9 (2)
Tb1—O3—C2—O4	171.1 (2)	O4^v^—Tb1—O1—Tb1^viii^	−36.98 (5)
Tb1—O3—C2—C2^iv^	−9.4 (4)	O4^vi^—Tb1—O1—Tb1^viii^	108.29 (11)
Tb1^x^—O4—C2—O3	−6.7 (5)	O4^vii^—Tb1—O1—Tb1^viii^	36.98 (5)
Tb1^iv^—O4—C2—O3	170.9 (2)	O4^iv^—Tb1—O1—Tb1^viii^	−108.29 (11)
Tb1^iv^—O4—C2—C2^iv^	−8.7 (4)	O4^v^—Tb1—O1—C1	143.02 (5)
Tb1^x^—O4—C2—C2^iv^	173.74 (18)	O4^vi^—Tb1—O1—C1	−71.71 (11)
O1^i^—Tb1—O1—Tb1^viii^	0.0	O4^vii^—Tb1—O1—C1	−143.02 (5)
O1^i^—Tb1—O1—C1	180.0	O4^iv^—Tb1—O1—C1	71.71 (11)
O1^i^—Tb1—O3—C2	54.2 (2)	O4^iv^—Tb1—O3—C2	9.75 (19)
O1—Tb1—O3—C2	−173.1 (2)	O4^vi^—Tb1—O3—C2	−21.7 (2)
O2^ii^—Tb1—O1—Tb1^viii^	180.0	O4^v^—Tb1—O3—C2	119.5 (2)
O2^ii^—Tb1—O1—C1	0.0	O4^vii^—Tb1—O3—C2	148.72 (19)

Symmetry codes: (i) *x*−1/2, *y*, −*z*+3/2; (ii) *x*−1/2, *y*, −*z*+1/2; (iii) *x*, −*y*+3/2, *z*; (iv) −*x*, −*y*+1, −*z*+1; (v) −*x*+1/2, −*y*+1, *z*+1/2; (vi) −*x*, *y*+1/2, −*z*+1; (vii) −*x*+1/2, *y*+1/2, *z*+1/2; (viii) *x*+1/2, *y*, −*z*+3/2; (ix) *x*+1/2, *y*, −*z*+1/2; (x) −*x*+1/2, −*y*+1, *z*−1/2.

### Hydrogen-bond geometry (Å, º)

**Table:**

*D*—H···*A*	*D*—H	H···*A*	*D*···*A*	*D*—H···*A*
C1—H1···O2^xi^	0.93	2.15	3.051 (5)	164

Symmetry code: (xi) *x*+1/2, −*y*+3/2, −*z*+1/2.
