# Supplementary material for: Crystal structure of a mixed-ligand terbium(III) coordination polymer containing oxalate and formate ligands, having a three-dimensional fcu topology
Source: Acta Crystallogr E Crystallogr Commun. 2016 Jan 1;72(Pt 1):87–91. doi: 10.1107/S205698901502397X (PMC4704756; doi:10.1107/S205698901502397X)
Supplement: Supplementary file 4 [file e-72-00087-Isup4.docx]

Supporting information

A three-dimensional fcu topology terbium(III) coordination polymer containing oxalate and formate mixed ligands

**Kittipong Chainok^a^*, Phailyn Khemthong^b^, Filip Kielar^b^ and Yan Zhou^c^**

^a^Department of Physics, Faculty of Science and Technology, Thammasat University, Phahon Yothin Road, Khlong Luang, Pathum Thani, 12120, Thailand
^b^Department of Chemistry, Faculty of Science, Naresuan University, Mueang, Phitsanulok, 65000, Thailand
^c^Department of Chemistry, The Hong Kong University of Science and Technology, Clear Water Bay, Kowloon, Hong Kong

Correspondence email: kc@tu.ac.th

1. Selected bond lengths and bond angles (Å, °) for (I).

| Tb1−O1 | 2.417 (3) | Tb1−O4^v^ | 2.437 (1) |
| --- | --- | --- | --- |
| Tb1−O1^i^ | 2.478 (3) | Tb1−O4^vi^ | 2.465 (1) |
| Tb1−O2^iii^ | 2.437 (3) | Tb1−Tb1^i^ | 3.8309 (2) |
| Tb1−O3 | 2.416 (1) |  |  |
|  |  |  |  |
| O1−Tb1−O1^ii^ | 133.65 (7) | O3−Tb1−O2^iii^ | 70.35 (5) |
| O1−Tb1−O2^iii^ | 100.16 (9) | O3−Tb1−O3^iv^ | 128.40 (10) |
| O1−Tb1−O3 | 77.72 (5) | O3−Tb1−O4^v^ | 126.90 (6) |
| O1^ii^−Tb1−O3 | 114.93 (5) | O3−Tb1−O4^vi^ | 72.19 (6) |
| O1−Tb1−O4^vi^ | 65.01 (6) | O3−Tb1−O4^vii^ | 66.88 (6) |
| O1^ii^−Tb1−O4^vii^ | 64.53 (6) | O3−Tb1−O4^viii^ | 132.53 (6) |
| O1−Tb1−O4^v^ | 144.49 (4) | O4^v^−Tb1−O4^vi^ | 140.95 (3) |
| O1^ii^−Tb1−O4^vi^ | 76.57 (6) | O4^v^−Tb1−O4^vii^ | 66.94 (8) |
| O2^iii^−Tb1−O1^ii^ | 126.19 (9) | O4^vi^−Tb1−O4^vii^ | 100.09 (6) |
| O2^iii^−Tb1−O4^v^ | 71.16 (7) | O4^vi^−Tb1−O4^viii^ | 66.08 (8) |
| O2^iii^−Tb1−O4^vi^ | 141.92 (5) | Tb1−O1−Tb1^i^ | 103.00 (9) |
|  |  | Tb1^vii^−O4−Tb1^ix^ | 102.79 (6) |

Symmetry codes: (i) *x* + 1/2, *y*, −*z* + 3/2; (ii) *x* − 1/2, *y*, −*z* + 3/2; (iii) *x* − 1/2, *y*, −*z* + 1/2; (iv) *x*, −*y* + 3/2, *z*; (v) −*x*, *y* + 1/2, −*z* + 1; (vi) –*x* + 1/2, −*y* + 1, *z* + 1/2; (vii) −*x*, −*y* + 1, −*z* + 1; (viii) –*x* + 1/2, *y* + 1/2, *z* + 1/2; (ix) –*x* + 1/2, −*y* + 1, *z* − 1/2.

1. Hydrogen-bond geometry (Å, °).

| *D*−H···A | *D*−H | H···A | *D*···A | *D*−H···A |
| --- | --- | --- | --- | --- |
| O1−H1*B*···O2^i^ | 0.93 | 2.15 | 3.051 (5) | 164 |

Symmetry codes: (i) *x* + 1/2, −*y* + 3/2, −*z* + 1/2.


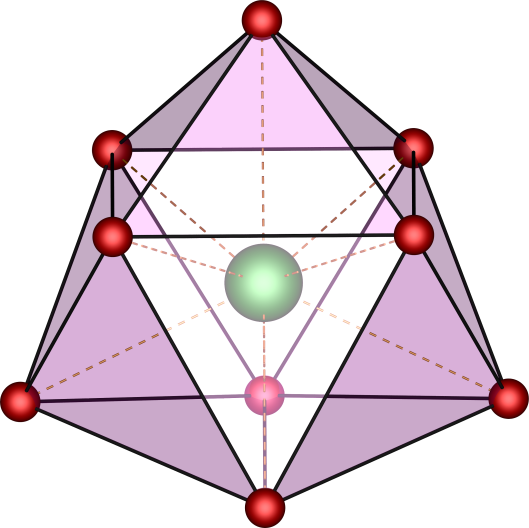


1. The coordination polyhedron of the Tb(III) ion in (I).


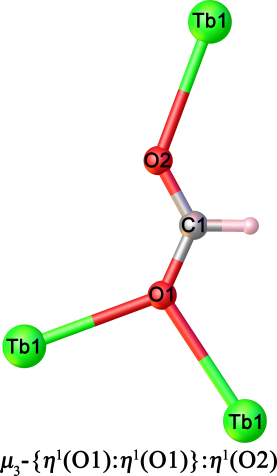

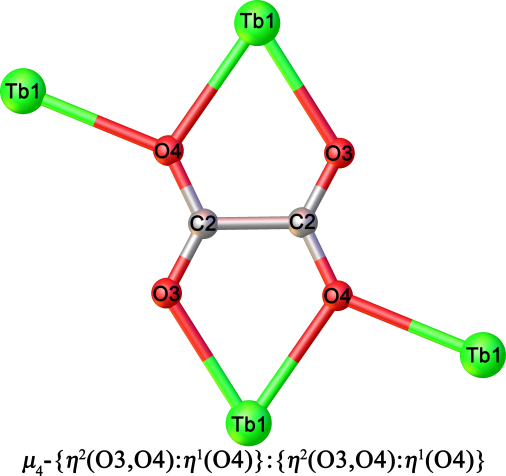


1. Coordination modes of formate and oxalate ligands observed in (I).


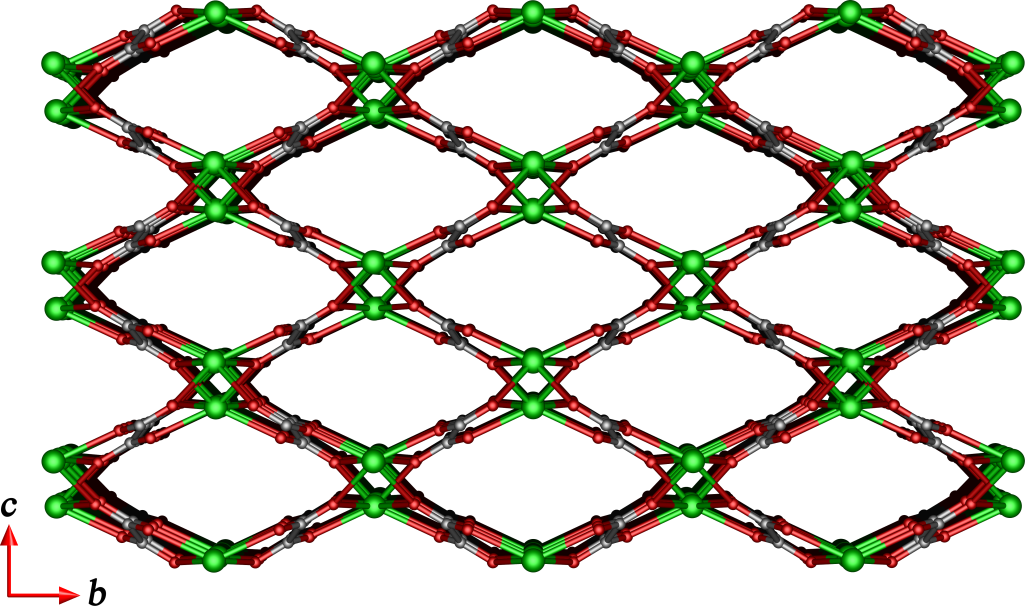


1. A perspective view of 3D terbium-oxalate open-framework for (I) along the *a* axis.


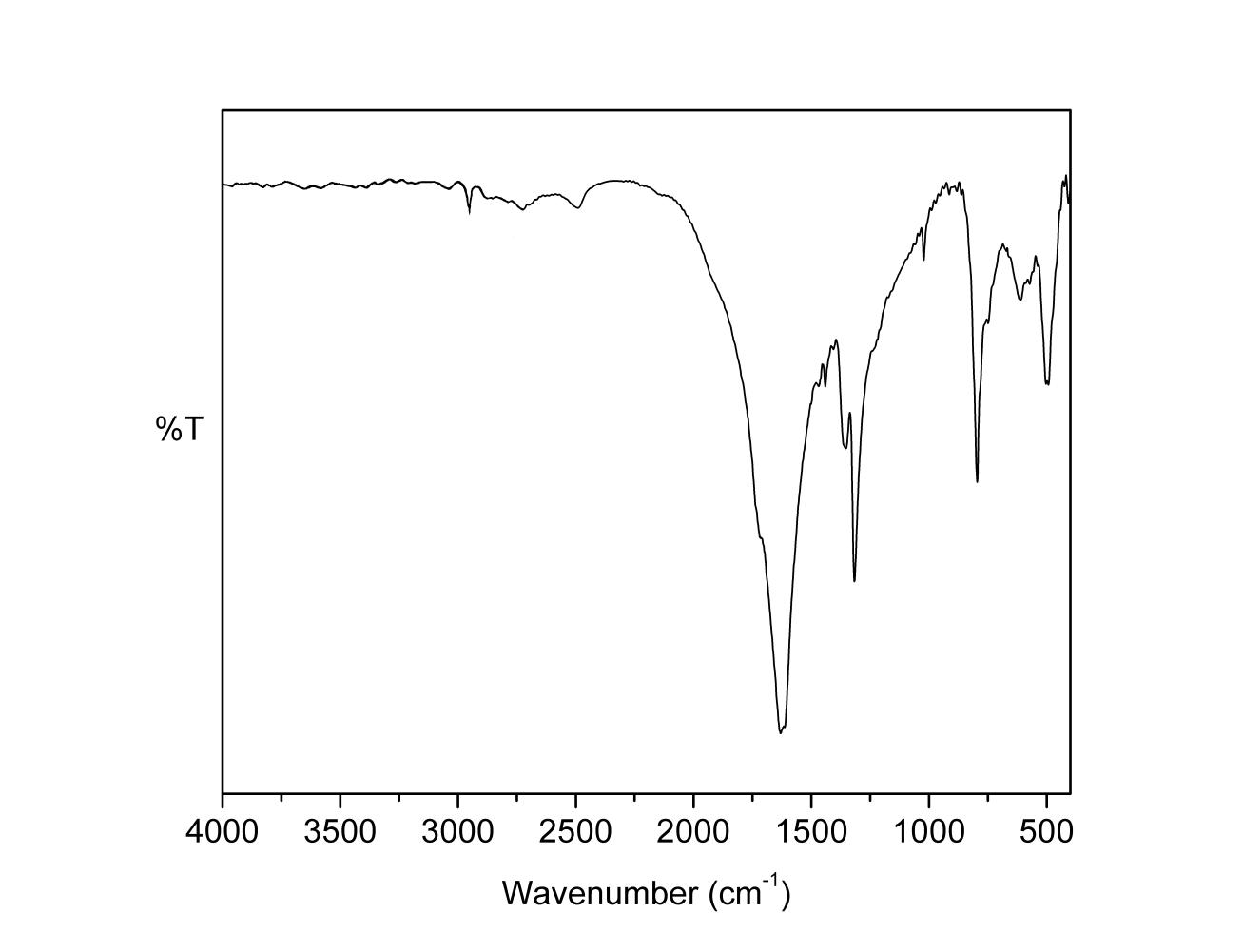


1. The IR spectrum of (I).


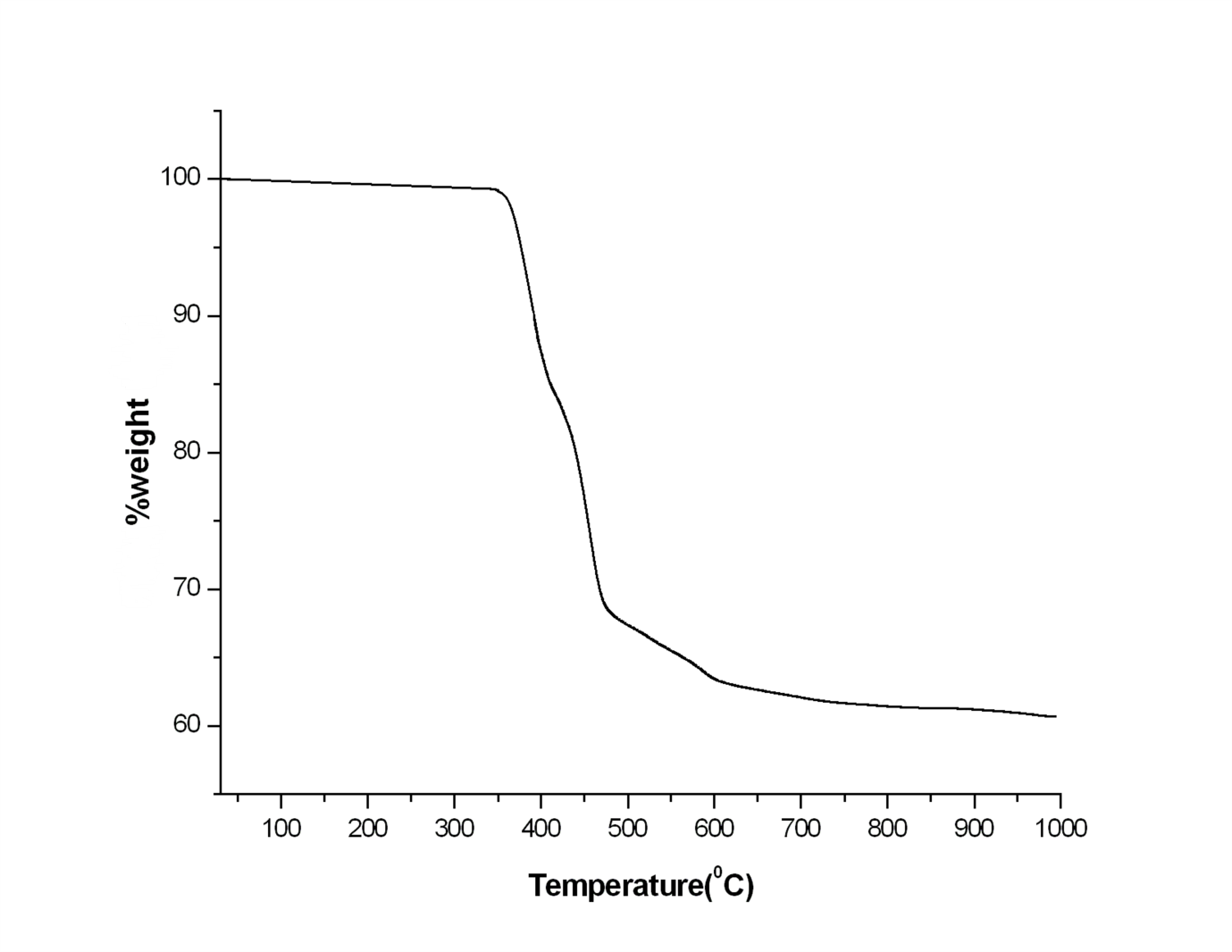


1. The TGA curve of (I).


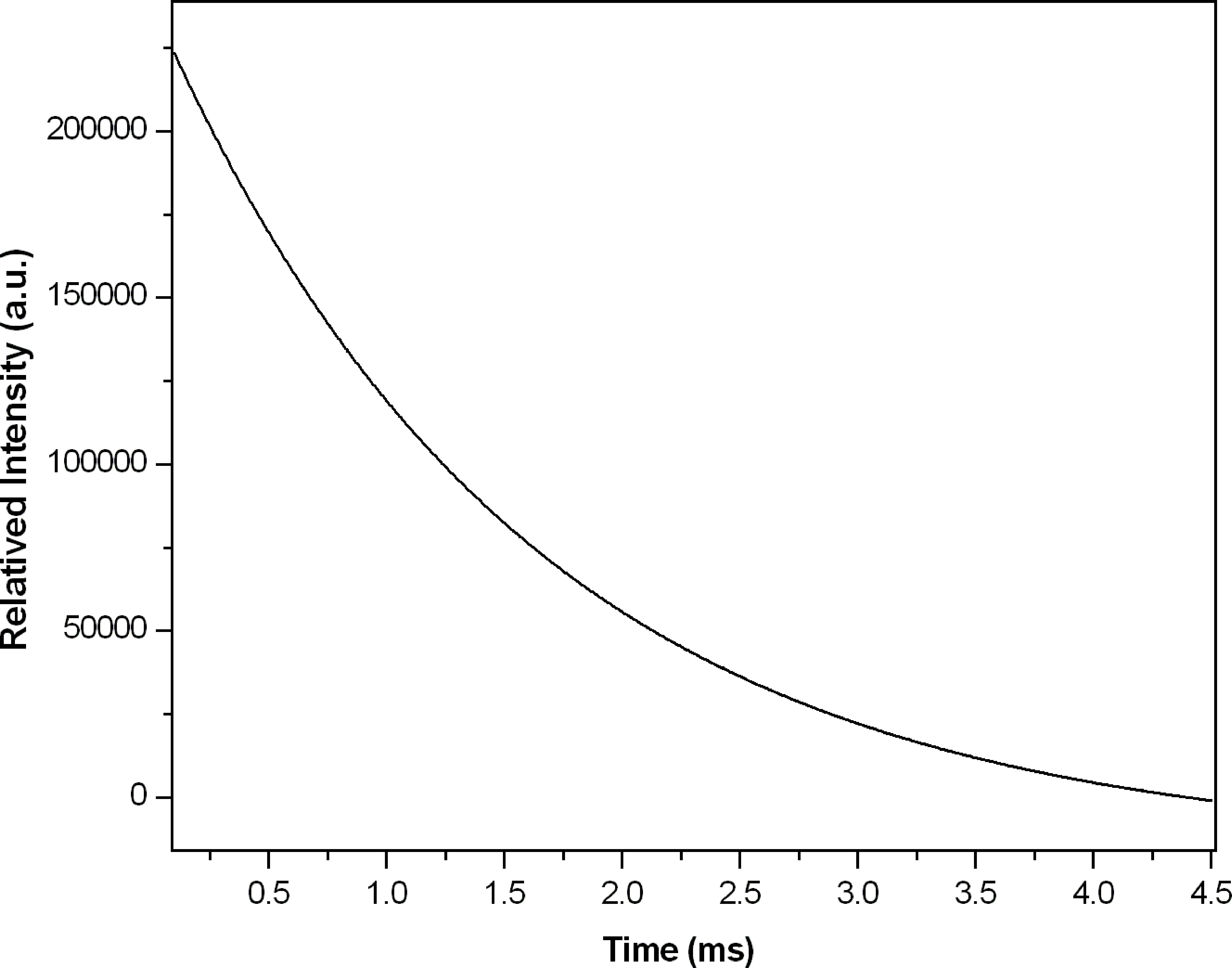


1. The emission intensity decays of (I).
